# HOPIS: Hybrid Omnidirectional and Perspective Imaging System for Mobile Robots

**DOI:** 10.3390/s140916508

**Published:** 2014-09-04

**Authors:** Huei-Yung. Lin, Min-Liang. Wang

**Affiliations:** Department of Electrical Engineering and Advanced Institute of Manufacturing with High-Tech Innovation, National Chung Cheng University, 168 University Road, Min-Hsiung, Chiayi 621, Taiwan; E-Mail: jw122huang@gmail.com

**Keywords:** robot vision, optical sensing, hybrid imaging geometry

## Abstract

In this paper, we present a framework for the hybrid omnidirectional and perspective robot vision system. Based on the hybrid imaging geometry, a generalized stereo approach is developed via the construction of virtual cameras. It is then used to rectify the hybrid image pair using the perspective projection model. The proposed method not only simplifies the computation of epipolar geometry for the hybrid imaging system, but also facilitates the stereo matching between the heterogeneous image formation. Experimental results for both the synthetic data and real scene images have demonstrated the feasibility of our approach.

## 1. Introduction

In the past few decades, the imaging geometry for perspective cameras has been well studied, first by the photogrammetry and then by the computer vision community. Multiple view relations between perspective cameras have also been established based on projective geometry [1]. Thus, recent research on geometric image formation is gradually moving toward the construction of catadioptric imaging models, which combines lenses and mirrors to increase the field of view [2,3]. The implementation with the so-called “omnidirectional camera” quickly gained popularity in mobile robot applications, mainly because of its 360° field of view [4,5]. Epipolar geometry and camera calibration for various types of catadioptric imaging models are also extensively investigated [6–8]. Although an omnidirectional camera is capable of capturing images of extremely large scenes, it suffers from low and non-uniform image resolution.

To increase the applicability of omnidirectional cameras, many researchers have proposed camera networks consisting of catadioptric and perspective sensing devices [9]. These approaches combine the advantages of the 360° field of view of omnidirectional cameras and high-resolution imaging from conventional cameras [10]. They are thus widely adopted in visual servoing [11,12], mobile robots for navigation [13,14] and localization [15,16] applications. This type of hybrid camera configuration can provide global and local vision simultaneously, but it also poses challenges for the imaging geometry and camera system calibration. One major issue is whether these two classes of cameras can be represented by the same imaging model, say the perspective projection model, which will then greatly facilitate the succeeding tasks, such as correspondence matching and 3D scene reconstruction. Unfortunately, there is still no single camera projection model that can be used to represent these heterogeneous imaging systems.

Due to the reflection nature of the omnidirectional cameras, the central catadioptric projection has to be processed by a two-step mapping via a sphere. It is thus not possible to have a unique and undistorted perspective image to represent the scene captured by an omnidirectional camera. Consequently, a concise computational model for multiple view geometry is not available for the hybrid omnidirectional and perspective configuration. The important geometric properties, such as the fundamental matrix, cannot be directly calculated in the 2D image space. In other words, the hybrid fundamental matrix for the perspective and omnidirectional camera pair cannot be directly estimated, even if the two cameras are fully calibrated.

To deal with the problem of mixing catadioptric and perspective cameras, Sturm analyzed the relationship between the multi-view images captured by para-catadioptric, perspective or affine cameras [17]. The fundamental matrices and planar homographies were derived in the lifted surface [18]. Chen *et al.* presented a three-step approach for hybrid camera network calibration [19]. The catadioptric camera was first calibrated using vanishing points; the perspective camera calibration was then carried out based on several derived 3D points. Cagnoni *et al.* developed a hybrid omnidirectional-pinhole sensor and derived the relationship between the two cameras using a surrounding calibration pattern box [20]. However, no theoretic imaging formation of the mixed camera model was addressed in their work. Chen and Yang proposed a homography-based image registration technique for the perspective and omnidirectional views [10]. Under the planar surface assumption, the image correspondences can be obtained without camera calibration. A similar technique was also developed by Adorni *et al.* using the inverse perspective transform between the omnidirectional and perspective images [21].

In this work, we are interested in the development of a hybrid omnidirectional and perspective imaging system (HOPIS) for mobile robot applications. In addition to the construction of the hybrid imaging geometry, we also present a unifying model to reduce the computational complexity of the hybrid camera system. Our objective is not to formulate the fundamental matrix of mixed view pairs from catadioptric and perspective cameras, but to study the generalized stereo matchingfor mixtures of different central projection systems. Consequently, stereo matching can be carried out using available techniques with rectified standard stereo image pairs.

The concept of the virtual image plane is introduced to simplify the imaging relations between the conventional and omnidirectional cameras. Unlike the previous work, such as [22,23], where the virtual images were generated via a straightforward image warping technique without taking the camera parameters into account, we propose virtual imaging formation based on the perspective projection model. The 3D reconstruction is thus feasible using the hybrid image pair. The proposed method not only establishes the epipolar geometry of the hybrid imaging system, but also facilitates the stereo matching between the heterogeneous image formation. We have shown that, for the non-degenerate cases, image rectification for any given perspective viewpoint is always possible from the catadioptric images. Thus, a generalized stereo imagecan be constructed from the hybrid of omnidirectional and perspective imaging.

The rest of this paper is organized as follows. Section 2 describes the imaging models of the perspective and catadioptric cameras. The generalized stereo of the hybrid imaging system via the virtual image plane is described in Section 3. In Section 4, we present the calibration technique for the perspective and catadioptric cameras. Experimental results are provided in Section 5, followed by the performance analysis of the system in Section 6. Finally, Section 7 concludes the paper and discusses some possible directions for future work.

## 2. Hybrid Omnidirectional and Perspective Imaging System

**Figure 1.**
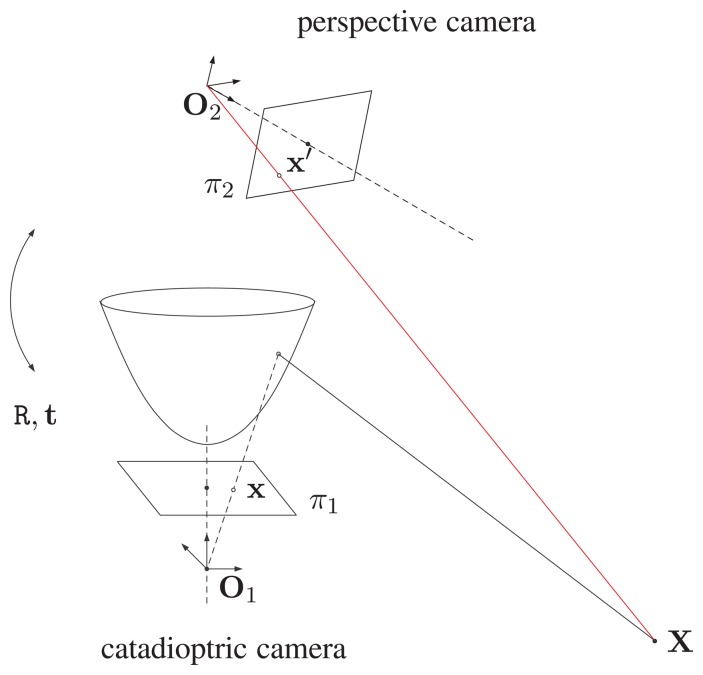
The proposed hybrid omnidirectional and perspective imaging system (HOPIS). It consists of a conventional camera and a catadioptric camera with a hyperboloidal mirror.

The proposed hybrid imaging system (HOPIS) consists of a conventional camera and a catadioptric camera with a hyperboloidal mirror, as shown in Figure 1.

These two types of cameras possess the property of single viewpoint projection, which is an essential condition for a unifying geometric representation. In this section, we describe the imaging model and calibration of both cameras, followed by the point correspondence relation between the two cameras.

### 2.1. Perspective Camera Model

For a perspective or pinhole camera, the relationship between a 3D point X̃ = (*X*,*Y*, *Z*)^⊤^ and the corresponding 2D image point x̃ = (*x*,*y*)^⊤^ can be written as:

(1) x=PX

where X and x are the 3D and image points represented by homogeneous four-vector and three-vector, respectively. The 3 × 4 homogeneous matrix P, which is unique up to a scale factor, is called the perspective projection matrix of the camera.

For the purpose of camera dissection and calibration, the perspective projection matrix can be further decomposed into the intrinsic camera parameter matrix and the relative pose of the camera, *i.e.*,

(2) P=K[R|t]

The 3 × 3 matrix R and 3 × 1 vector t are the relative orientation and translation with respect to the world coordinate system, respectively. The intrinsic parameter matrix K of the camera is a 3 × 3 matrix and usually modeled as:

(3) $$\text{K}=\left[\begin{array}{lll} f_{x} & \gamma & u_{0}\\ 0 & f_{y} & \upsilon_{0}\\ 0 & 0 & 1 \end{array}\right]$$

where (*u*_0_, *υ*_0_) is the principal point of the camera (the intersection of the optical axis with the image plane), γ is a skew parameter related to the characteristic of the CCD array and *f_x_* and *f_y_* are scale factors in the x and y directions of the image sensor.

### 2.2. Omnidirectional Camera Model

The imaging models and calibration techniques for the omnidirectional cameras have been extensively investigated since panoramic image formation was introduced [24]. For central catadioptric cameras with a single viewpoint, the most popular imaging geometry is represented by a two-step projection via a unit sphere [25]. Based on this projection model, Barreto and Araujo use an image with at least three lines to calibrate the catadioptric cameras [26]. Ying and Hu present a calibration method using geometric invariants of lines and spheres [7]. Mei and Rives take the lens distortion into account and calibrate the omnidirectional camera using planar grids [27].

Using the unit sphere projection model, a 3D scene point X is first projected to a point X*_s_* on the unit sphere located at the origin O of the unifying catadioptric projection model. The point X*_s_* is then projected perspectively via a projection center O_c_ located inside the unit sphere to the image plane of the lens camera. For a general catadioptric imaging system, the optical axis of the lens camera is aligned with the line determined by the two centers of projections O and O_c_. Let the point X_s_ be represented by (*X_s_* *,Y_s_, Z_s_*, 1)^⊤^ in homogeneous coordinates, then the projection of the 3D scene point X on the image plane is given by:

(4) $$\text{m}={\left(X_{s},Y_{s},Z_{s}+\xi \right)}^{⊤}$$

where ξ ∈ [0,1] is the distance between the two projection centers, O and O*_c_*. The image point x of the 3D scene point X can finally be obtained by incorporating the internal camera project matrix K*_c_*, as in Equation (3), *i.e.*,

(5) $$\text{x}={\text{K}}_{c}\text{m}$$

In this unifying catadioptric projection model, the camera projection center and coordinate system are defined in terms of the unit sphere. The extrinsic parameters of the omnidirectional camera are the rotation matrix R and the translation vector t with respect to the world coordinate system. The intrinsic parameters include the effective focal length, image center and skew factor of the perspective projection. The implementation of omnidirectional camera calibration with both the intrinsic and extrinsic parameters can be found, for example, in [27,28]. The latter is used for our HOPIS calibration, as described in Section 4.

### 2.3. Hybrid Imaging System

Figure 1 illustrates the point correspondence relation between the omnidirectional and perspective images. Suppose the centers of projection of the catadioptric and perspective cameras are O_1_ and O_2_, respectively. Given a 3D point X viewable to both cameras, its projections to the image planes π_1_ and π_2_ are fully described by the intrinsic and extrinsic parameters of the hybrid imaging system.

Suppose a light ray from the 3D point X reflected by the hyperbolic mirror of the catadioptric camera intersects the image plane π_1_ at point x. The point correspondence x′ on the image plane π_2_ can be modeled by a coordinate transformation R, t from O_1_ to O_2_. If the focal length or sensor resolution of the cameras are different, then an additional internal transformation for the perspective camera needs to be carried out. Thus, the 3D point X can be uniquely determined by the point correspondences x and x′.

## 3. Generalized Stereo Model via Virtual Image Plane

**Figure 2.**
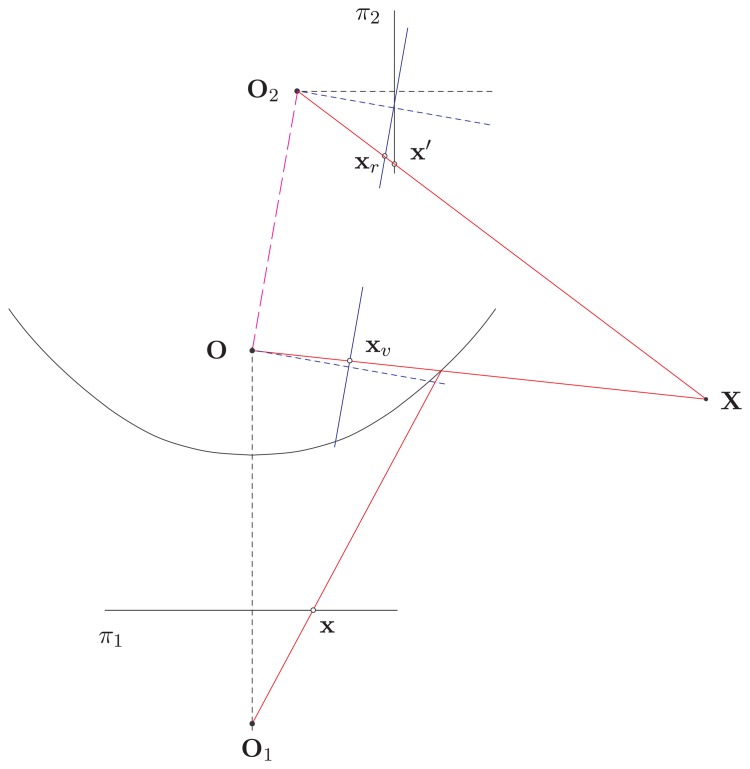
The proposed HOPIS configuration. O_1_ and O_2_ are the optical centers of the catadioptric and perspective cameras, respectively. O is the effective viewpoint of the catadioptric image formation. A 3D scene point X is projected to x and x′ on the omnidirectional and perspective images, respectively.

To simplify the 3D reconstruction formulation for a pair of hybrid omnidirectional and perspective images, a generalized stereo model using the concept of the virtual image plane is proposed. The objective is to rectify the hybrid image pairs to form the fronto-parallel stereo ones, which possess the property of parallel epipolar lines. For the proposed HOPIS configuration, as shown in Figure 2, a virtual camera is constructed associated with the omnidirectional camera, such that the optical axis is perpendicular to the baseline between the catadioptric and perspective cameras. By warping and transforming the omnidirectional and perspective images to the common virtual image plane, a rectified stereo image pair can be derived.

### 3.1. Construction of Virtual Cameras

For a single-viewpoint catadioptric imaging system, the effective viewpoint is located at the focus of the quadric surface behind the reflection mirror. Let O be the sphere center of the unifying catadioptric projection model and O_2_ be the projection center of the perspective camera. To rectify the hybrid omnidirectional and perspective image pair for stereo matching, the virtual cameras with the same effective viewpoints as the hybrid imaging system can be constructed with the stereo baseline OO_2_ as follows.

Let the coordinate system O be the common reference frame of the hybrid camera system, and the orientation and translation of the perspective camera O_2_ are R and t, respectively. Rotate the perspective camera, such that the image scanlines (*i.e.*, along the *y*-axis) are parallel to the translation vector t. The associated rotation matrix R′ is then applied to the effective catadioptric viewpoint O to create a virtual camera with the same focal length and orientation as the perspective camera O_2_. To summarize, the image rectification for the hybrid imaging system involves the rotation transformations R′ and R^−1^R′ for the viewpoints O and O_2_, respectively. Finally, the common focal length of the cameras can be adjusted to increase the overlapping scene of the rectified image pair.

It should be noted that some configurations are not physically realizable due to the non-overlapping scenes captured by the perspective and omnidirectional cameras. For example, the combination of an upward perspective camera and a downward omnidirectional camera with coincident optical axes does not have a common field of view. If we consider the special case that O_2_ lies on the line determined by O and O_1_, both the rotation transformations R and R′ are the identity matrix. In this case, only the virtual perspective image for the catadioptric camera has to be constructed, and no additional image rectification has to be carried out.

### 3.2. Virtual Image Generation

Image rectification for the perspective camera can be performed with a straightforward linear warping technique provided that the intrinsic and extrinsic parameters of the camera are available [29]. The generation of virtual perspective images from an omnidirectional image, however, is not a simple one-to-one linearmapping. As described in Section 2.2, the catadioptric image formation can bemodeled by a unifying projection with a two-step linear mapping via a unit sphere. Thus, the virtual images can be synthesized by back-projecting the rays from the omnidirectional image.

**Figure 3.**
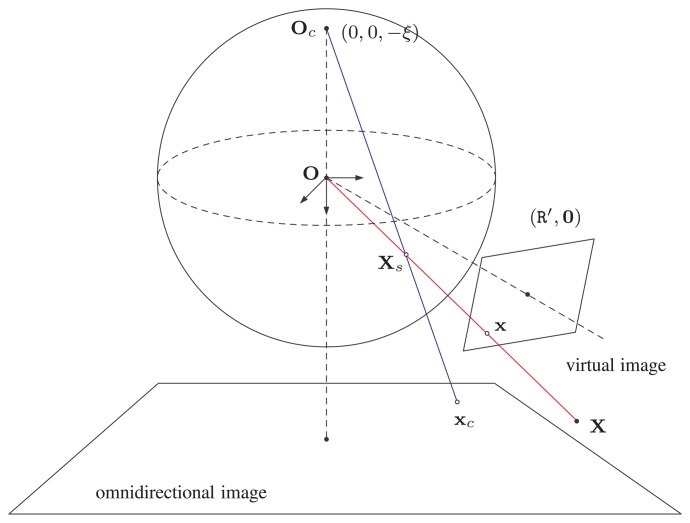
Catadioptric image formation model via a unit sphere used for virtual camera construction. The origin of the common coordinate frame O is the projection center of both catadioptric and virtual cameras.

As shown in Figure 3, the center of the unit sphere O is the effective viewpoint of the catadioptric camera. For image rectification with the rotation matrix R′, the projection matrix of the virtual camera is:

(6) $${\text{P}}_{\upsilon }={\text{K}}_{\upsilon }\left[{\text{R}}^{\prime }|0\right]$$

where K*_υ_* is the intrinsic parameter matrix. The projection of a 3D scene point X to the image point x on the virtual image plane is given by:

(7) $$\text{x}={\text{P}}_{\upsilon }\text{X}=\left[{\text{K}}_{\upsilon }{\text{R}}^{\prime }|\text{0}\right]\text{X}=\left[{\text{K}}_{\upsilon }{\text{R}}^{\prime }|\text{0}\right]\left(\begin{array}{c} \tilde{\text{X}}\\ 1 \end{array}\right)$$

or:

(8) $$\text{x}={\text{K}}_{\upsilon }{\text{R}}^{\prime }\tilde{\text{X}}$$

where X̃ is the inhomogeneous representation of X. Thus, we have:

(9) $$\tilde{\text{X}}={\text{R}\prime }^{-1}{\text{K}}_{\upsilon }^{-1}\text{x}$$

and

(10) $${\text{X}}_{s}\sim \text{X}=\left(\begin{array}{c} {\text{R}\prime }^{-1}{\text{K}}_{\upsilon }^{-1}\text{x}\\ 1 \end{array}\right)$$

where X*_s_* can also be represented by:

(11) $${\text{X}}_{s}={\left({\tilde{\text{X}}}_{s},1\right)}^{⊤}={\left(x_{s},y_{s},z_{s},1\right)}^{⊤}$$

Now, the mapping of X̃*_s_* onto the omnidirectional image plane via the projection center O_c_ is given by:

(12) $${\text{x}}_{c}={\text{P}}_{c}{\text{X}}_{s}={\text{A}}_{c}{\text{R}}_{c}[\text{I}|-{\text{O}}_{c}]{\text{X}}_{s}$$

where A_c_ and R_c_ are the camera matrix and the extrinsic orientation of the perspective projection, respectively. Since both A_c_ and R_c_ are the intrinsic parameters of the catadioptric camera, Equation (12) can be rewritten as:

$$\begin{array}{c} {\text{x}}_{c}={\text{K}}_{c}\left(\begin{array}{c} x_{s}\\ y_{s}\\ z_{s}+\xi \sqrt{x_{s}^{2}+y_{s}^{2}+z_{s}^{2}} \end{array}\right)\\ ={\text{K}}_{c}({\tilde{\text{X}}}_{s}+\xi ||{\tilde{\text{X}}}_{s}||{\text{e}}_{3}) \end{array}$$

or:

(13) $${\text{x}}_{c}={\text{K}}_{c}(R\prime^{-1}{\text{K}}_{\upsilon }^{-1}\text{x}+\xi \left\|{\text{R}\prime }^{-1}{\text{K}}_{\upsilon }^{-1}\right\|{\text{e}}_{3})$$

where e_3_ = (0, 0,1)^⊤^, K_c_ = A_c_R_c_ and O_c_ = (0, 0, −ξ).

Equation (13) establishes the one-to-one correspondence between x and x*_c_*. Thus, the virtual image with given camera matrix *K_υ_* and orientation R′ can be synthesized from the omnidirectional image. Furthermore, the rectified image can then be used with the perspective camera O_2_, as described in Section 2.3, for stereo matching and 3D reconstruction.

### 3.3. Triangulation from the Hybrid Image Pair

The image formation of the hybrid camera system consists of the projections of a 3D scene point to the conventional and omnidirectional cameras. Suppose that the extrinsic parameters of the perspective camera and the virtual camera generated from the catadioptric camera in the world coordinate system are (R*_p_*, t*_p_*) and (R*_υ_*, t*_υ_*), respectively. Then, the transformation (R, t) between the two camera coordinate frames is given by:

$$\begin{array}{l} {\text{R}=\text{R}}_{p}{\text{R}}_{\upsilon }^{-1}\\ \text{t}={\text{t}}_{\upsilon }-{\text{R}}_{\upsilon }^{-1}{\text{t}}_{p} \end{array}$$

The projection relations of 3D scene points to both cameras can be derived as follows.

Let *x_p_* and *x_υ_* be the projections of a 3D point X on the image plane of the perspective and the virtual camera associated with the catadioptric system, respectively. Suppose that the projection error of the 3D scene point is modeled, then X can be derived as the midpoint of the line segment perpendicular to the rays back projected from both camera centers and passing through the image points x*_p_* and x*_υ_*, respectively. Given the rotation and translation between the perspective and omnidirectional cameras, these two rays are represented by *ax_p_* and t + bR^⊤^x*_υ_*, where *a*, *b* ∈ ℝ, and the 3D scene point X can be derived by solving a system of linear equations [30].

It should be noted that, the above triangulation is not based on image rectification and can be performed with any relative orientation and translation (R, t) between the two cameras. Suppose that a correspondence matching (x*_p_*, x*_o_*) of the perspective and omnidirectional image pair is identified; it is possible to choose a suitable rotation matrix Rv for the construction of a virtual image from the catadioptric camera. Thus, a vergence stereo configuration can be easily achieved to increase the overlapping region observed from the perspective and virtual cameras.

## 4. HOPIS Calibration

Camera calibration for the proposed hybrid omnidirectional and perspective imaging system is to derive the intrinsic parameters of both cameras and the relative pose between the cameras. For the initial system calibration, a checkerboard pattern is placed at a location viewable to both cameras. Tsai's method is carried out to estimate the camera matrix *K_p_* and extrinsic parameters (R*_p_*, t*_p_*) of the perspective camera [31]. The orientation and position of the camera are calculated relative to the world coordinate frame set on the calibration pattern.

To calibrate the omnidirectional camera, the technique presented by Mei and Rives is adopted [27]. Several omnidirectional images captured with different positions and orientations of the checkerboard pattern are used to estimate the intrinsic parameter matrix K*_c_* and the relative poses with respective to different pattern coordinate frames. The exterior orientation and translation of the camera coordinate system, (R*_o_*, t*_o_*), is the one relative to the same calibration pattern used for the perspective camera. Note that the origin of the omnidirectional camera coordinate system is the sphere center of the unifying projection model.

### 4.1. Rotation and Translation between the Cameras

In the initial system calibration, the rotation and translation between the omnidirectional and perspective cameras can be obtained through the common world coordinate frame. This relative orientation and position within the hybrid camera system, however, might not be constant over time for some applications. An active HOPIS configuration with a PTZ camera can be used for surveillance, robot navigation and human computer interaction, *etc*. In these cases, auto-calibration for orientation update has practical uses and is highly desirable.

From the decomposition of the essential matrix:

(14) E=[t]×R

the relative orientation and the direction of translation between a pair of perspective cameras can be derived [32]. Thus, if both coordinate frames of the constructed virtual camera and the associated catadioptric camera are aligned, the rotation and translation (R, t) can be obtained by computing the essential matrix. Now, suppose the intrinsic parameter matrices K*_υ_* and K*_p_* of the virtual and perspective cameras are available from the initial calibration. Then, from the relation:

(15) $$\text{E}={{\text{K}}_{\upsilon }}^{⊤}{\text{FK}}_{p}$$

where F is the fundamental matrix of the stereo image pair, the essential matrix can be derived from eight image point correspondences [33].

From Equations (14) and (15), the orientation and translation can be derived by:

(16) $$\left[\text{t}\right]\times \text{R}={{\text{K}}_{\upsilon }}^{⊤}{\text{FK}}_{p}$$

Using the point correspondences of the two perspective images. In the hybrid stereo image pair, however, only the correspondences between the omnidirectional and perspective images are directly accessible. Suppose a point correspondence x*_c_* ↔ x′ is identified, where x*_c_* and x′ belong to the omnidirectional and perspective images, respectively. The corresponding point x on the virtual camera can be derived from Equation (13) with R′ = I, *i.e.*,

(17) $${\text{x}}_{c}={\text{K}}_{c}(K_{\upsilon }^{-1}\text{x}+\xi ∥K_{\upsilon }^{-1}\text{x}∥{\text{e}}_{3})$$

Thus, the rotation and translation (R, t) is obtained up to a scale provided that all intrinsic camera matrices are calibrated. The unknown scale factor for the translation t can be determined using a fixed distance between two 3D scene points.

### 4.2. Feature Matching for the Hybrid Image Pair

**Figure 4.**
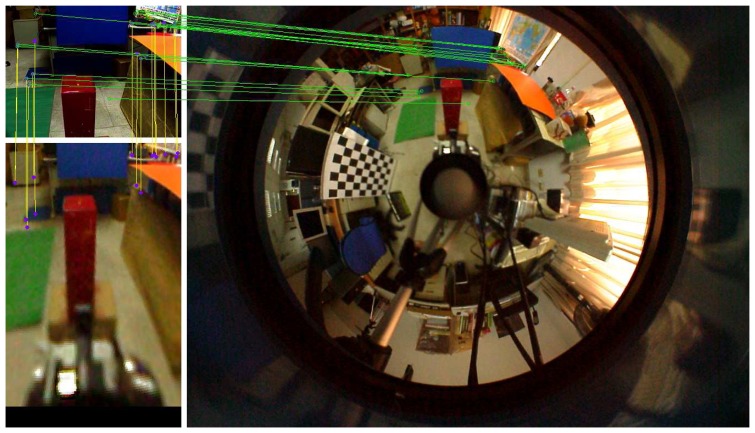
The omnidirectional and perspective images captured in a real-world scene. The green lines indicate the correspondences of SIFT features via direct matching. The yellow lines represent the correspondence matching between the perspective and virtual images. The result shows that the feature matching on the virtual-perspective image pair is better than the direct matching on the omnidirectional-perspective image pair.

To find the point correspondences between the omnidirectional and perspective images, the SIFT descriptor is adopted for feature matching [34]. Since unwarping the omnidirectional image to a panoramic form generally increases the searching range, the correspondence matching is carried out on the original hybrid stereo image pair. The search region is further reduced with the knowledge of an approximate orientation of the perspective camera. For example, the searching range on the omnidirectional image can generally be restricted to less than a quarter of the image, depending on the field-of-view of the perspective camera. An example of the feature correspondence matching between the omnidirectional and perspective images is illustrated in Figure 4.

## 5. Experiments

**Figure 5.**
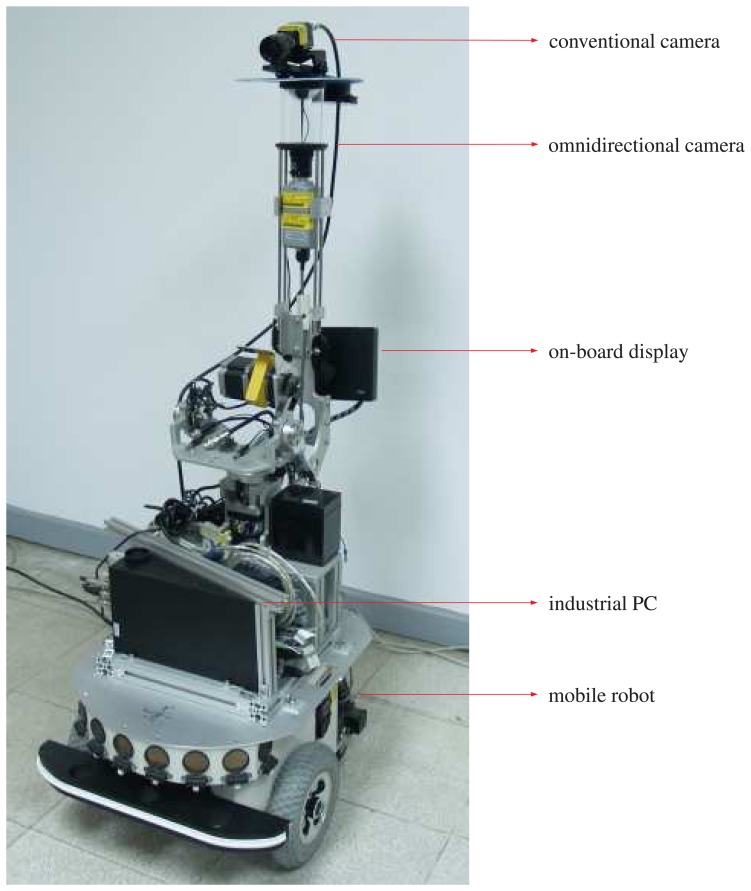
The mobile robot platform and HOPIS used in the real scene experiments. The conventional camera is capable of changing the viewpoint through the motor controlled pan-tilt motion.

**Figure 6.**
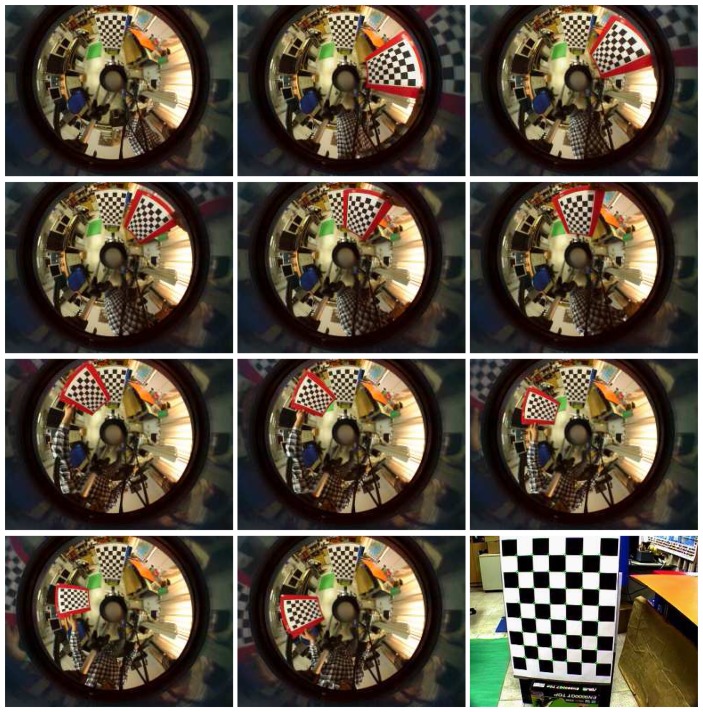
The images captured by the omnidirectional and perspective cameras are used for system calibration. The checkerboard pattern shown in the perspective and the first omnidirectional image served as the world coordinate frame of the camera system.

**Figure 7.**
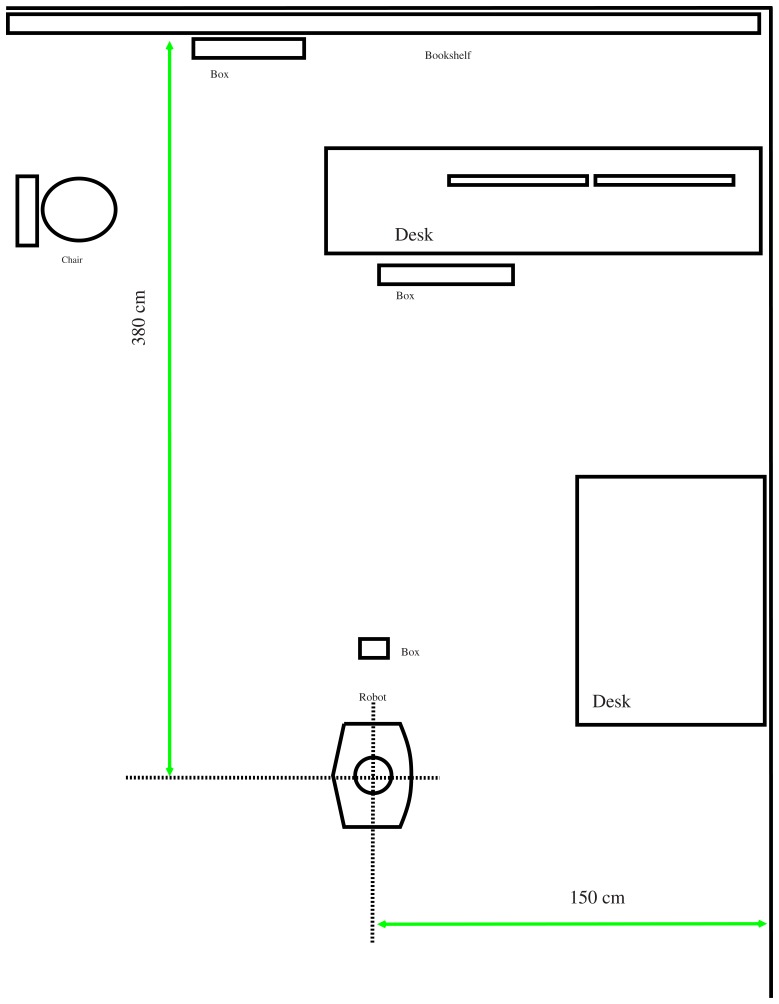
The experimental environment.

**Figure 8.**
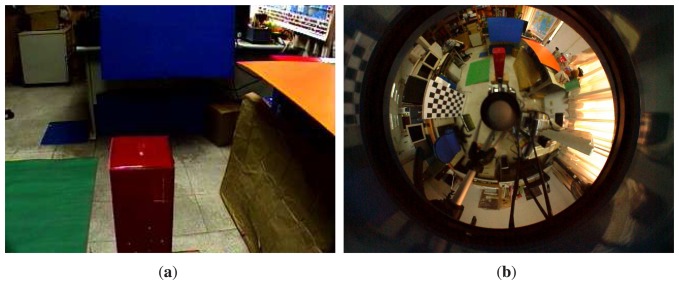
The hybrid omnidirectional and perspective image pair used for the first experiment. The image resolutions are 320 × 240 and 1024 × 768, respectively. (**a**) Perspective image; (**b**) omnidirectional image.

We have performed a number of experiments to assess the effectiveness of the proposed generalized stereo model for the hybrid imaging system. The hybrid camera system, which consists of a Watec-221s analog camera and a SONY DFW-X710 digital camera with an attached hyperbolic mirror, is mounted on a mobile robot, as shown in Figure 5. The image resolutions of the cameras are 320 × 240 and 1024 × 768, respectively. All images were acquired with white-balancing and auto-exposure. The perspective camera equipped with a Tamron 12VM612T lens provided a field of view of 30.4° × 23.1°. The intrinsic and extrinsic parameters of the camera system are obtained as described in Section 4. Figure 6 shows the captured omnidirectional and perspective images used for system calibration. The experimental environment and the acquired hybrid stereo image pair are shown in Figures 7 and 8, respectively.

**Figure 9.**
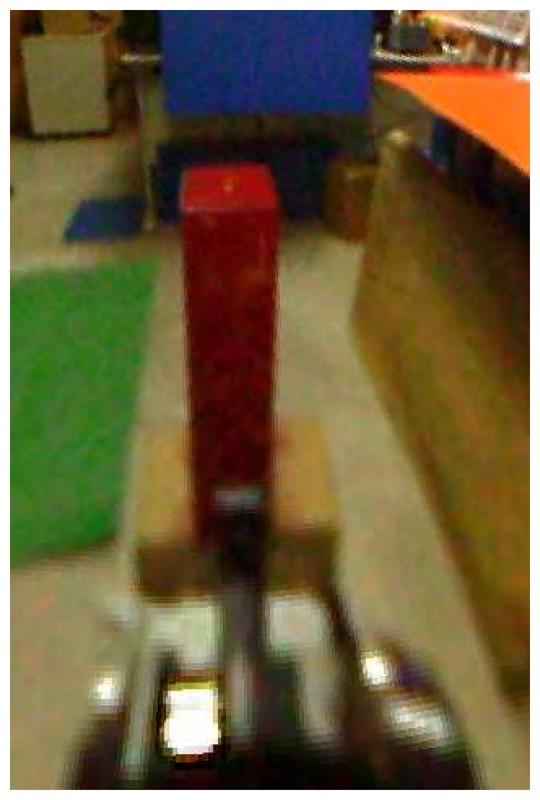
The virtual image generated from the omnidirectional image, Figure 8b. The image resolution is set as 320 × 480 for large overlap with the perspective image, Figure 8a.

In developing the generalized stereo model by constructing a virtual image from the omnidirectional image, the algorithm described in Section 3 is carried out. Since the field of view of the virtual camera can be set arbitrarily for any fixed focal length, the virtual image can be created with different image sensor sizes. As shown in Figure 5, the perspective camera is placed above the catadioptric camera in our HOPIS configuration. Thus, the resolution of the virtual image is set as 320 × 480, with the extension in the vertical direction to increase the overlap with the perspective image. Figure 9 shows the virtual image generated from the omnidirectional image (see Figure 8b) with the same viewing direction as the perspective image (see Figure 8a).

**Figure 10.**
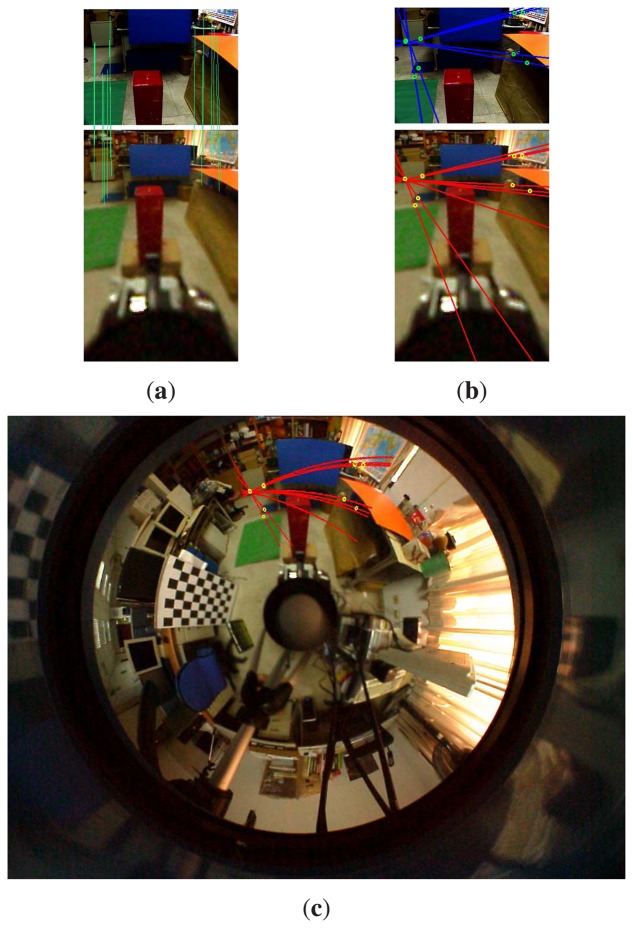
The epipolar geometry of the hybrid omnidirectional and perspective image pair. Figure 10a illustrates the SIFT correspondences between the perspective and virtual images. The epipolar line pairs between the perspective and virtual images are shown in Figure 10b. The corresponding epipolar lines on the omnidirectional image are illustrated in Figure 10c. (**a**) Feature matching; (**b**) epipolar lines; (**c**) the corresponding epipolar lines on the omnidirectional image.

Figure 10 illustrates the epipolar geometry of the hybrid omnidirectional and perspective imaging system. Consider the case that the virtual camera is constructed to have the same orientation as the perspective camera. Figure 10a illustrates the correspondence matching between the perspective image and the virtual image derived from the omnidirectional image. The rotation between the two cameras estimated by these point correspondences is very close to the calibration result. If their optical axes are not perpendicular to the stereo baseline, then the epipoles will lie on the image planes, as illustrated in Figure 10b. Although the epipolar lines can be easily derived from the epipolar geometry of a stereo rig, they correspond to the quadric curves in the omnidirectional image, as shown in Figure 10c. Thus, the stereo matching is greatly simplified on the virtual and perspective image pair, even if they are not rectified to the fronto-parallel configuration.

**Figure 11.**
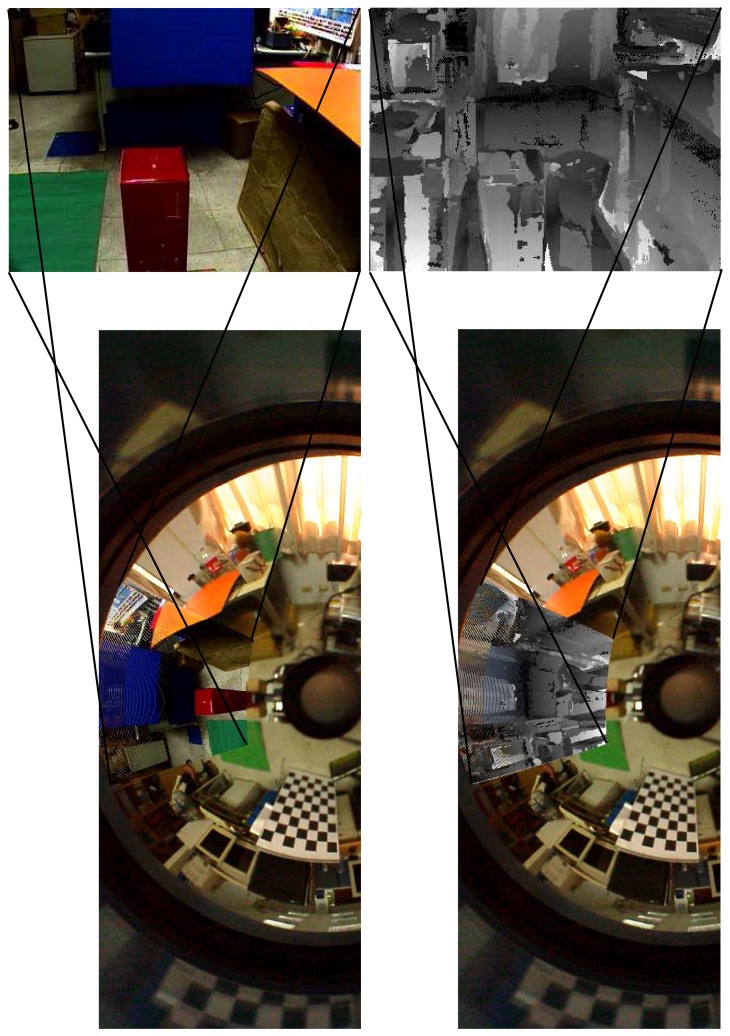
The matching results on the perspective and omnidirectional image pair for the first experiment. The perspective image and the associated depth map is projected to the common region in the omnidirectional view for illustration.

**Figure 12.**
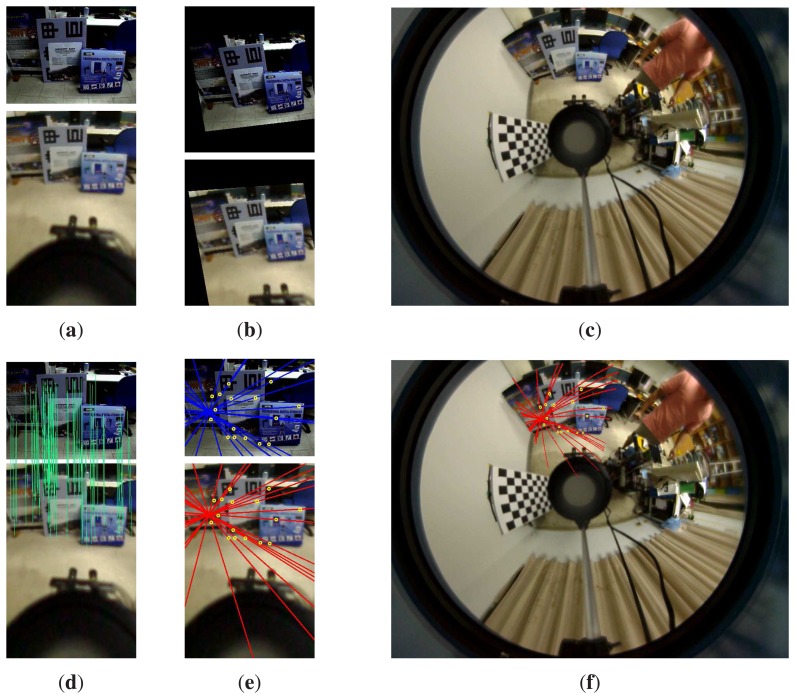
The second experimental result. The captured omnidirectional and perspective images are shown in (**c**) and (**a**) (top). (**a**) (bottom) The generated virtual image. The rectified stereo image pair is shown in (**b**). (**d**) The SIFT correspondences between the perspective and virtual images. The epipolar geometry of the hybrid stereo image pair is illustrated in (**e**) and (**f**).

Figure 11 illustrates the matching results on the perspective and omnidirectional image pair. A specific region of interest captured by the perspective camera (as shown on the top) is used for stereo matching and depth reconstruction. The depth map is then transferred to the common region in the omnidirectional view for global depth perception. As shown on the bottom of Figure 11, the misalignment on the region of interest is due to the parallax between the omnidirectional and perspective cameras. Figure 12 shows another experiment with the input hybrid stereo image pair, epipolar geometry, image rectification and disparity maps (see the captions for more details).

## 6. Evaluation on Feature Matching

Similar to the conventional stereo vision systems, it is important to understand the performance of correspondence matching with respect to various camera parameter settings of the proposed hybrid imaging system; more specifically, given a fixed translation and orientation between the omnidirectional and perspective cameras, how to achieve better feature detection and matching results by adjusting their focal lengths and image resolutions. In this work, the performance evaluation on correspondence matching is based on the detection of SIFT features in both cameras. While the intrinsic parameters can be directly changed for the perspective camera, those associated with the omnidirectional camera are only accessible in terms of the virtual images.

To evaluate the effect of focal length on the correspondence matching, the synthetic images are generated with various focal lengths for both the perspective and virtual cameras. The detection and matching of the SIFT features for different focal lengths are tabulated in Table 1. Table 2 tabulates the correspondence matching of the SIFT features for various focal lengths and image resolutions of the virtual camera. The same focal length is used for both the perspective and virtual cameras. For each image resolution and focal length, the number of correspondences is calculated by averaging the results from ten captured and generated stereo image pairs. It is clear that the number of detected features and the processing time increases with the image resolution. However, as shown in the table, higher resolution does not guarantee better correspondence matching results. This is mainly due to the severe image distortion of high resolution warping from the omnidirectional image.

For the perspective camera, the number of detected features increases with the focal length, mainly due to the zoom-in effect on textured patterns. The virtual images, on the other hand, have steady feature extraction results for all focal lengths, since they are all synthesized from the same omnidirectional image. For the correspondence matching between the perspective and virtual images, the same focal length settings for both cameras generally provide the best matching results, as shown on the diagonal of the table.

**Figure 13.**
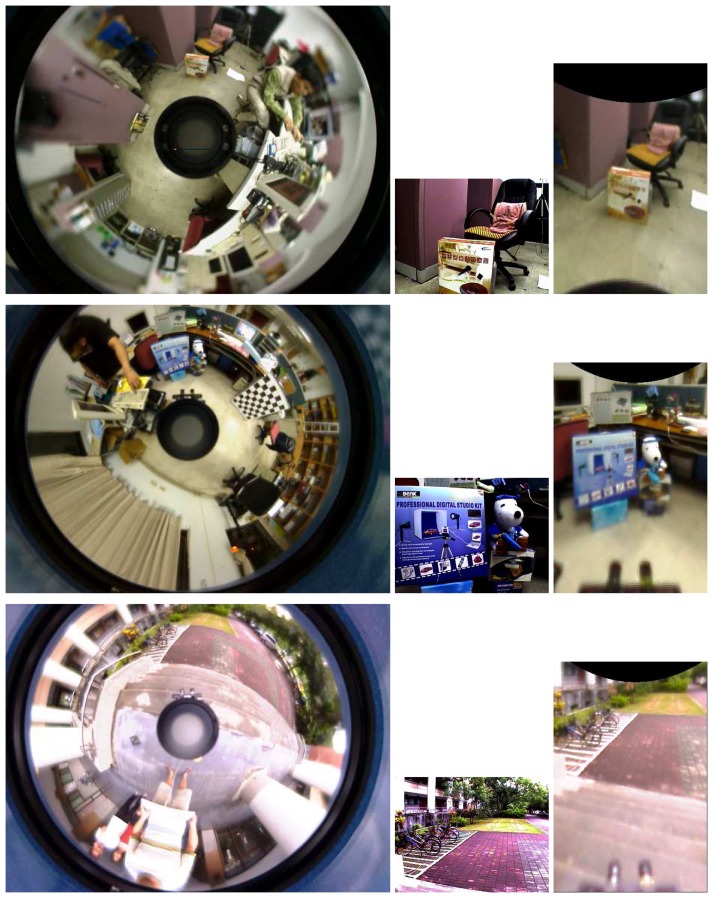
Some test images used for performance comparison, as shown in Table 3.

It is well known that the feature matching between two heterogeneous image pairs is a difficult task, even using scale-invariant feature descriptors. Thus, it is interesting to investigate the improvement on the feature matching results if the virtual image plane is introduced. We have examined the averages of matched features, mismatched features, error matching rate and computation time of the omnidirectional-perspective and the proposed virtual-perspective image pairs. The performance comparison of SIFT matching results using five indoor and two outdoor scenes (three of them are shown in Figure 13) is presented in Table 3. It can be seen that the error matching rate and computation time of the proposed technique are both much lower than the conventional full range search between the perspective and omnidirectional image pair.

## 7. Conclusion

In this work, we have presented a generalized stereo approach for the hybrid imaging system consisting of a conventional and an omnidirectional camera. The proposed technique provides the robot vision system with the capability of omnidirectional surveillance and 3D reconstruction. It can be used for mobile robot applications, such as obstacle detection, with the derived 3D information and vision-guided navigation using the omnidirectional images [35]. The imaging formation of the hybrid camera system is formulated using a unifying projection model. The epipolar geometry of the hybrid omnidirectional and perspective image pair is simplified by the mapping via a virtual camera. With image rectification and reprojection, stereo matching between the heterogeneous images can be carried out using available techniques for standard image pairs. Thus, our approach is suited for depth recovery using the hybrid omnidirectional and perspective camera system. It is also possible to replace the conventional camera with a PTZ (pan-tilt-zoom) camera, making the region for depth recovery more flexible [36]. The experimental results are presented for both the simulated data and real scene images.

## Figures and Tables

**Table 1.** SIFT feature detection and matching for the perspective camera (p.c.) and virtual camera (v.c.) with various focal lengths.

p.c.	10	20	30	40	50	60	70	80	Total Features
v.c.									
10	**57**	48	47	46	42	45	53	49	82
20	46	**63**	47	49	38	48	56	53	99
30	31	38	**50**	36	29	37	53	46	70
40	38	50	49	**63**	44	51	53	52	98
50	29	32	42	39	47	43	**51**	40	80
60	38	47	52	50	51	**66**	62	60	89
70	40	43	53	46	41	58	**70**	55	86
80	44	52	45	48	44	56	68	**75**	112
Total Features	74	80	93	79	77	95	108	110	

**Table 2.** SIFT feature detection and correspondence matching between the hybrid stereo image pairs for various focal lengths and image resolutions. The HOPIS correspondences are obtained from the average of ten image pairs. The SIFT features are those detected in the virtual images. The execution time is in seconds.

Cell Size	Image Resolution	HOPIS Correspondences			SIFT Features			Execution Time		
		f = 6 mm	f = 9 mm	f = 12 mm	f = 6 mm	f = 9 mm	f = 12 mm	f = 6 mm	1 = 9 mm	f = 12 mm
0.2	1600 × 2400	21.8	22.4	15.1	531	268	206	9.9	9.2	8.9
0.25	1280 × 1920	23.8	20.8	16.8	456	245	184	7.2	6.9	6.5
0.5	640 × 960	24.5	22.6	14.0	346	168	131	3.2	2.7	2.6
1	320 × 480	26.2	25.9	14.5	358	245	122	2.3	2.0	1.7
2	160 × 240	28.4	26.1	13.0	237	225	162	1.9	1.8	1.6
3	106 × 160	21.0	24.7	12.3	141	149	152	1.5	1.6	1.5
4	80 × 120	12.8	22.1	13.5	93	124	122	1.4	1.3	1.4
5	64 × 96	9.9	18.1	17.1	61	86	90	1.3	1.2	1.3

**Table 3.** Comparison of the SIFT feature correspondences between the direct matching of the omnidirectional-perspective and the proposed virtual-perspective image pairs. The execution time is in seconds.

Hybrid Image Pair	Omnidirectional-Perspective		Virtual-Perspective	
	Omnidirectional	Perspective	Virtual	Perspective
Image resolution	1024 × 768	320 × 240	320 × 480	320 × 240
Average SIFT features	1565.14	440.42	374.85	440.42
Average matching features	27.7		24.85	
Average error matching	2.428		1.714	
Average error matching rate	8.76%		**6.89%**	
Average computation time	5.37		**1.87**	

## References

[1] Hartley R.I., Zisserman A. (2004). Multiple View Geometry in Computer Vision.

[2] Baker S., Nayar S. (1999). A Theory of Single-Viewpoint Catadioptric Image Formation. Int. J. Comput. Vis..

[3] Yamazawa K., Yagi Y., Yachida M. Omnidirectional imaging with hyperboloidal projection.

[4] Yagi Y. (1999). Omnidirectional Sensing and Its Applications. IEICE Trans. Inf. Syst..

[5] Menegatti E., Pretto A., Scarpa A., Pagello E. (2006). Omnidirectional vision scan matching for robot localization in dynamic environments. IEEE Trans. Robot..

[6] Svoboda T., Pajdla T. (2002). Epipolar Geometry for Central Catadioptric Cameras. Int. J. Comput. Vis..

[7] Ying X., Hu Z. (2004). Catadioptric camera calibration using geometric invariants. IEEE Trans. Pattern Anal. Mach. Intell..

[8] Smadja L., Benosman R., Devars J. (2006). Hybrid Stereo Configurations through a Cylindrical Sensor Calibration. Mach. Vis. Appl..

[9] Gandhi T., Trivedi M.M. (2006). Reconfigurable omnidirectional camera array calibration with a linear moving object. Image Vis. Comput..

[10] Chen D., Yang J. (2005). Image Registration with Uncalibrated Cameras in Hybrid Vision Systems.

[11] Mariottini G.L., Prattichizzo D. (2008). Image-Based Visual Servoing with Central Catadioptric Cameras. Int. J. Rob. Res..

[12] Tahri O., Mezouar Y., Andreff N., Martinet P. (2009). Omnidirectional Visual-Servo of a Gough-Stewart Platform. IEEE Trans. Robot..

[13] Menegatti E., Maeda T., Ishiguro H. (2004). Image-based memory for robot navigation using properties of omnidirectional images. Robot. Auton. Syst..

[14] Spacek L., Burbridge C. (2007). Instantaneous robot self-localization and motion estimation with omnidirectional vision. Robot. Auton. Syst..

[15] Murillo A., Sagues C., Guerrero J., Goedeme T., Tuytelaars T., Gool L.V. (2007). From omnidirectional images to hierarchical localization. Robot. Auton. Syst..

[16] Scaramuzza D., Fraundorfer F., Pollefeys M. (2010). Closing the loop in appearance-guided omnidirectional visual odometry by using vocabulary trees. Robot. Auton. Syst..

[17] Sturm P. Mixing catadioptric and perspective cameras.

[18] Sturm P., Barreto J. (2008). General Imaging Geometry for Central Catadioptric Cameras. Computer Vision-ECCV 2008.

[19] Chen X., Yang J., Waibel A. Calibration of a hybrid camera network.

[20] Cagnoni S., Mordonini M., Mussi L., Adorni G. Hybrid Stereo Sensor with Omnidirectional Vision Capabilities: Overview and Calibration Procedures.

[21] Adorni G., Mordonini M., Cagnoni S., Sgorbissa A. Omnidirectional stereo systems for robot navigation.

[22] Moldovan D., Wada T. A calibrated pinhole camera model for single viewpoint omnidirectional imaging systems.

[23] Peri V., Nayar S. Generation of Perspective and Panoramic Video from Omnidirectional Video.

[24] Benosman R., Kang S., Faugeras O. (2001). Panoramic Vision: Sensors, Theory, and Applications.

[25] Geyer C., Daniilidis K. (2001). Catadioptric Projective Geometry. Int. J. Comput. Vis..

[26] Barreto J., Araujo H. (2005). Geometric properties of central catadioptric line images and their application in calibration. IEEE Trans. Pattern Anal. Mach. Intell..

[27] Mei C., Rives P. Single View Point Omnidirectional Camera Calibration from Planar Grids.

[28] Scaramuzza D., Martinelli A., Siegwart R. A Toolbox for Easily Calibrating Omnidirectional Cameras.

[29] Fusiello A., Trucco E., Verri A. (2000). A compact algorithm for rectification of stereo pairs. Mach. Vis. Appl..

[30] Trucco E., Verri A. (1998). Introductory Techniques for 3-D Computer Vision.

[31] Tsai R. (1987). A Versatile Camera Calibration Technique for High-Accuracy 3D Machine Vision Metrology Using Off-the-Shelf TV Cameras and Lenses. IEEE Trans. Robot. Autom..

[32] Longuet-Higgins H. (1981). A Computer Algorithm for Reconstructing a Scene from Two Projections. Nature.

[33] Hartley R. (1997). In defense of the eight-point algorithm. IEEE Trans. Pattern Anal. Mach. Intell..

[34] Lowe D.G. (2004). Distinctive Image Features from Scale-Invariant Keypoints. Int. J. Comput. Vis..

[35] Wang M.L., Lin H.Y. (2011). An Extended-HCT Semantic Description for Visual Place Recognition. Int. J. Rob. Res..

[36] Yu M.S., Wu H., Lin H.Y. A visual surveillance system for mobile robot using omnidirectional and PTZ cameras.

